# Complete genome sequence of the probiotic *Bifidobacterium adolescentis* strain iVS-1

**DOI:** 10.1128/MRA.00541-23

**Published:** 2023-11-09

**Authors:** Katherine Chacón-Vargas, Mallory J. Van Haute, Isabella M. K. Kessinger, Kaylee A. McClain, Shara R. P. Yumul, Chloe M. Christensen, Zachery T. Lewis, Thomas A. Auchtung

**Affiliations:** 1 Synbiotic Health, Inc, Lincoln, Nebraska, USA; Wellesley College, Wellesley, Massachusetts, USA

**Keywords:** *Bifidobacterium adolescentis*, iVS-1, lactose, folate, GABA

## Abstract

*Bifidobacterium adolescentis* iVS-1 is a human-isolated strain known to possess several probiotic properties. Here, its genome was completely sequenced to examine genes associated with lactose metabolism and other potentially beneficial traits, such as the production of folate and gamma-aminobutyric acid (GABA).

## ANNOUNCEMENT

*Bifidobacterium adolescentis* is a species in the phylum Actinomycetota that is common in the gastrointestinal tracts of primates (1), including humans (2). Animal and *in vitro* studies suggest that *B. adolescentis* strains can provide beneficial functions, including reducing anxiety and inflammation (3, 4), protecting against or improving recovery from several diseases (e.g., references 5, 6), and metabolism of prebiotics such as xylooligosaccharides (7), galactooligosaccharides (GOS) (8), and arabinoxylan (9).

*B. adolescentis* iVS-1 was originally isolated from the fecal sample of a human that consumed increasing amounts of GOS (8, 10). In a clinical trial, iVS-1 improved intestinal barrier integrity (11) and persisted after treatment in several subjects (12). A draft genome was previously sequenced (NCBI Assembly ASM82986v1); we now describe the complete genome.

Cells were grown to late exponential phase in 300 mL reinforced clostridial medium (RCM; BD Difco), pelleted by centrifugation, and shipped to CD Genomics (Shirley, NY, USA), where total DNA was extracted using a DNeasy UltraClean Microbial Kit (Qiagen), sheared to 10 kb using a g-TUBE (Covaris), constructed into a SMRTbell (v2.0) library, and sequenced on a PacBio Sequel II (v2.0 chemistry). Reads were corrected and adapter sequences were filtered by Canu (v1.6), resulting in 416,354 subreads with an average length of 9,546 bp (N_50_ = 10,333 bp), mean depth of 1,473, and 100% coverage. A single scaffold was assembled with Flye (v2.8.3-b1695) using default parameters.

The *B. adolescentis* iVS-1 genome is 2,306,390 bp, with a GC content of 59.62%, which is similar to other sequenced *B. adolescentis* strains (2.09–2.40 Mbp and 59.2–59.9% GC; CP028341.1, CP053072.1, CP117956.1, CP023005.1, CP024959.1, CP007443.1, CP010437.1, CP047129.1, AP009256.1, and LR698990.1). Genome annotation with RAST (13) showed a total of 1,885 protein coding genes, 13 rRNA, 56 tRNA, and 1 CRISPR region. Confirmation that iVS-1 clusters with other *B. adolescentis* strains was obtained with PATRIC (14), which aligned predicted protein sequences to those in the PGFam database (15) with MUSCLE (16), and analyzed alignments with RaxML (17). Default parameters were used for all software unless otherwise specified.

**Fig 1:**
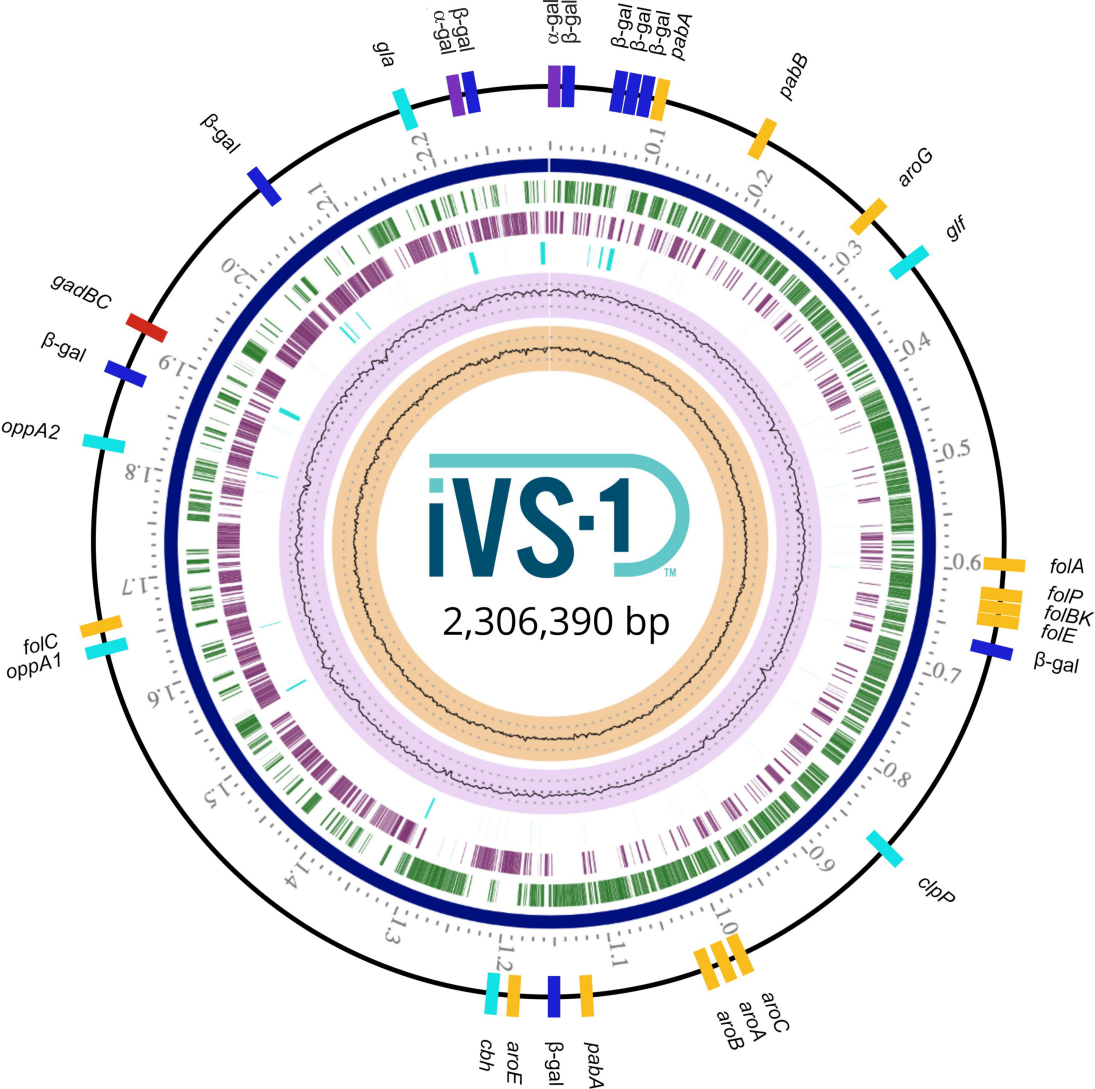
Genome map of *Bifidobacterium adolescentis* iVS-1: From the inner to outer circle are GC skew (tan), GC content (light purple), non-CDS features (aqua), CDS reverse strand (purple), CDS forward strand (green), tick marks (Mb), and genes of probiotic interest (GABA production, red; folate production, yellow; degradation of β-galactosides, dark blue; degradation of α-galactosides, purple; bile, osmotic, and acid stress resistance, light blue). The base figure was generated with BV-BRC Genome View (23).

Genes potentially conferring benefits to the host were examined (Fig. 1). The iVS-1 genome has two α-galactosidase genes for metabolism of carbohydrates such as raffinose (18) and nine different β-galactosidase genes. The iVS-1 genome also encodes *cbh*, *clpP*, *gla*, *glf*, and *oppA*, which may be important for resisting bile, osmotic, and/or acid stress (19), *fol* genes for *de novo* production of folate, and *aro* and *pab* genes for synthesis of the folate precursors chorismate and para-aminobenzoic acid (20, 21). Finally, iVS-1 encodes the genes to synthesize the neurotransmitter gamma-aminobutyric acid (GABA) via a glutamate decarboxylase (*gadB*) coupled with a glutamate/gamma-aminobutyrate antiporter (*gadC*) (22).

## Data Availability

The iVS-1 BioProject, BioSample, SRA, and GenBank complete assembled genome accession numbers are PRJNA954850, SAMN34156685, SRR24147545, and CP123050.
